# Tumor necrosis factor α, and agonist and antagonists of cannabinoid receptor type 1 and type 2 alter the immunophenotype of stem cells from human exfoliated deciduous teeth

**DOI:** 10.31744/einstein_journal/2023AO0405

**Published:** 2023-10-26

**Authors:** Marizia Trevizani, Laís Lopardi Leal, João Vitor Paes Rettore, Gilson Costa Macedo, Caio César de Souza Alves, Sandra Bertelli Ribeiro de Castro, Antônio Márcio Resende do Carmo, Silvioney Augusto da Silva, Carlos Magno da Costa Maranduba, Fernando de Sá Silva

**Affiliations:** 1 Instituto de Ciências Biológicas Universidade Federal de Juiz de Fora Juiz de Fora MG Brazil Instituto de Ciências Biológicas , Universidade Federal de Juiz de Fora , Juiz de Fora , MG , Brazil .; 2 Faculdade de Medicina do Mucuri Universidade Federal dos Vales do Jequitinhonha e Mucuri Teófilo Otoni MG Brazil Faculdade de Medicina do Mucuri , Universidade Federal dos Vales do Jequitinhonha e Mucuri , Teófilo Otoni , MG , Brazil .; 3 Universidade Federal de Juiz de Fora Governador Valadares MG Brazil Universidade Federal de Juiz de Fora , Governador Valadares , MG , Brazil .

**Keywords:** Endocannabinoids, Mesenchymal stem cells, Tooth, deciduous, Immunomodulation, Receptor, cannabinoid, CB1, Receptor, cannabinoid, CB2, Tumor necrosis factor-alpha

## Abstract

**Objective:**

To verify the involvement of the endocannabinoid system in the immunomodulatory profile of stem cells from human exfoliated deciduous teeth, in the presence or absence of TNF-α, and agonist and antagonists of CB1 and CB2.

**Methods:**

Stem cells from human exfoliated deciduous teeth were cultured in the presence or absence of an agonist, anandamide, and two antagonists, AM251 and SR144528, of CB1 and CB2 receptors, with or without TNF-α stimulation. For analysis of immunomodulation, surface molecules linked to immunomodulation, namely human leukocyte antigen-DR isotype (HLA-DR), and programmed death ligands 1 (PD-L1) and 2 (PD-L2) were measured using flow cytometry.

**Results:**

The inhibition of endocannabinoid receptors together with the proinflammatory effect of TNF-α resulted in increased HLA-DR expression in stem cells from human exfoliated deciduous teeth, as well as, in these cells acquiring an anti-inflammatory profile by enhancing the expression of PD-L1 and PD-L2.

**Conclusion:**

Stem cells from human exfoliated deciduous teeth respond to the endocannabinoid system and TNF-α by altering key immune response molecules.

## Highlights

Inhibition of endocannabinoid receptors and TNF-α led to an increase in HLA-DR, PD-L1, and PD-L2 levels in stem cells from human exfoliated deciduous teeth.

This study shows the interaction between mesenchymal stromal cells and the immune and endocannabinoid systems.

## Graphical abstract

**Figure:**
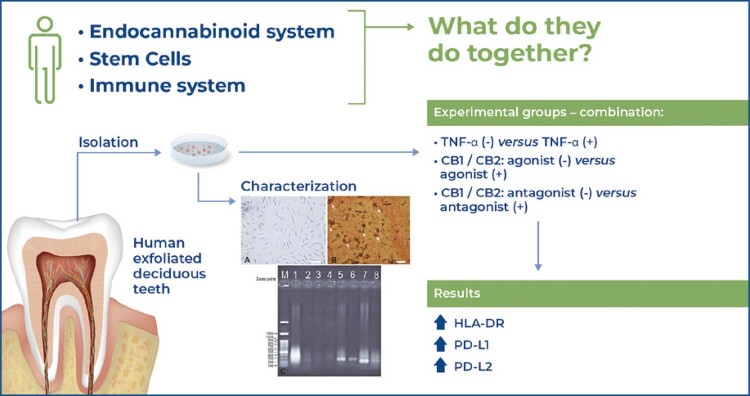

## INTRODUCTION

Mesenchymal stromal cells (MSCs) are multipotent adult stem cells derived from various tissues, which are capable of self-renewal and maintain their undifferentiated state after several replication cycles. ^( 1 - 12 )^ Besides regenerative properties, MSCs possess pronounced immunomodulatory capacity. These cells can exert their immunosuppressive effects by cell-cell contact and by secreting immunomodulatory molecules, such as indoleamine 2, 3-dioxygenase (IDO), transforming growth factor beta (TGF-β), and prostaglandin E2 (PGE2). ^( 3 , 5 , 8 , 11 - 25 )^

PD-L1, PD-L2, and HLA are important components of the immunomodulatory profile related to cell communication and signaling. ^( 26 - 29 )^ PD-L1 and PD-L2 are co-stimulatory factors that inhibit T helper cell activation, regulatory T cell (Treg) apoptosis, and cytokine secretion. ^( 30 - 32 )^ Mesenchymal stromal cells express and secrete PD-L1 and PD-L2 upon stimulation with interferon gamma (IFN-γ) and tumor necrosis factor alpha (TNF-α). ^( 30 , 33 )^ They exhibit low expression of HLA-DR, which can be increased under an inflammatory environment. ^( 34 )^

The endocannabinoid system (ECS) comprises a family of receptors and endogenous ligands, and the molecular machinery for their synthesis, which has been best explored in the central nervous system (CNS). ^( 35 )^ The main components of the ECS are as follows: two “classical” endocannabinoid ligands, namely N-arachidonoyl ethanolamine anandamide (AEA) and 2-arachidonoylglycerol (2-AG), and “classical” cannabinoid receptors, namely type 1 (CB1) and type 2 (CB2). ^( 35 - 44 )^ Besides the CNS, CB1 and CB2 have also been discovered in the immune system ^( 45 - 47 )^ and MSCs and participate in their regenerative and immunomodulatory processes. ^( 48 , 49 )^

Studies on the role of the ECS in MSCs have been scarce. Mesenchymal stromal cells can express all the components of the ECS related to immunomodulatory properties, survival pathways, and migration. These cells also produce endocannabinoids, such as AEA, 2-AG, and oleoylethanolamide (OEA). ^( 48 , 50 )^

## OBJECTIVE

To evaluate the immunomodulatory profile of stem cells from human exfoliated deciduous teeth in the presence of TNF-α, and agonist and antagonists of CB1 and CB2.

## METHODS

### Isolation of SHED

Stem cells from human exfoliated deciduous teeth (SHED) were isolated from a healthy volunteer and cultured according to Gronthos et al. ^( 1 )^ and Liu et al., ^( 51 )^ with some modifications. The dental pulp was dilacerated with a scalpel and then washed in Dulbecco’s modified Eagle medium: Nutrient mixture F-12 (DMEM/F12) (Invitrogen, USA) supplemented with 10% fetal bovine serum (FBS; LGC Biotecnologia, Brasil), 100U/mL penicillin, 100µg/mL streptomycin, 2mM L-glutamine, and 0.01mM non-essential amino acids (Invitrogen), until the release of cells. The isolated SHED were kept in 75cm ^2^ culture flasks in supplemented DMEM/F12 at 37 ^o^ C under 5% CO _2_ and expanded until 70% confluence.

The acquisition of teeth followed a declaration of consent signed by persons responsible for the minor. All proceedings were performed in accordance with the ethical standards of the local ethics committee (CAAE: 33905014.4.0000.5133; #003/2011). The samples were stored in the Biobank Human Genetics and Cell Therapy (CONEP B-030), *Universidade Federal de Juiz de Fora* .

### Molecular characterization of SHED

The expression of hematopoietic ( *CD34* and *CD45* ), mesenchymal ( *NES* and CD105), and embryonic ( *NANOG* and *OCT4* ) markers on SHED was analyzed using RT-PCR. Briefly, total RNA was extracted from cells using the RNeasy Mini kit (Qiagen, Dusseldorf, Germany), according to the manufacturer’s instructions. RNA quality was determined by concentration and purity of RNA by measuring absorbance at 260 and 280nm. Expression of the genes was analyzed on cDNA fragments obtained with the QIAGEN OneStep RT-PCR Kit (Qiagen) using the primers described in table 1 . The products were electrophoresed on 2% agarose gels, stained with ethidium bromide, and visualized with the Gel Logic 100 Imaging System transilluminator (Kodak, New Haven, USA), using Kodak 1D Software, v. 3.6.5 K2 (Kodak). The experiment was performed in triplicates.

**Table 1:** Primer sequences

Markers	*Primer* F (5’>3’)	*Primer* R (5’>3’)	*Amplicon (bp)*	Tm (°C)
Embryonic				
* OCT4*	ACTTCACTGCACTGTACTCCTCAG	AGGTTCTCTTTCCCTAGCTCCTC	158	60
* NANOG*	CTACCCCAGCCTTTACTCTTCCTAC	CTCTCCACAGTTATAGAAGGGACTG	217	60
Hematopoietic				
* CD34*	AACACCTAGTACCCTTGGAAGTACC	AACACTGTGCTGATTACAGAGGTC	177	60
* CD45*	GGACACAGAAGTATTTGTGACAGG	GAGAAGTTGTGGTCTCTGAGAAGTC	176	60
Mesenchymal				
* NES*	GGACCCTCCTAGAGGCTGAG	GTGAGGAGAGGGGAGTAGGG	168	60
* CD105*	TGCCACTGGACACAGGATAA	CCTTCGAGACCTGGCTAGTG	205	60
Controls				
* ACTB*	ATTAAGGAGAAGCTGTGCTACGTC	GATGGAGTTGAAGGTAGTTTCGTG	213	60
* GAPDH*	GAAGGTGAAGGTCGGAGTC	GAAGATGGTGATGGGATTTC	226	58

### Osteogenic differentiation of SHED

The differentiation capacity of SHED was assessed *in vitro* . The established lines were subjected to osteogenic differentiation. The cells were plated in 6-well plates at a density of 1 × 10 ^4^ cells/well. After 24 hours of culture in regular medium, the cells were washed, the medium in the wells was replaced with an osteogenic differentiation medium (DMEM-LG, 10% FBS, 1 × 10 ^-8^ M dexamethasone, 0.2 M L-ascorbic acid 2-phosphate, and 10mM β-glycerol phosphate), and the cells were maintained in culture for 14 days. All experiments were done in triplicates. At the end of the culture period, calcium deposition was evaluated using Alizarin Red S (Sigma-Aldrich, USA) staining, according to the manufacturer’s instructions. Images were obtained with an inverted microscope (Nikon TS100F, Japan) using 4× and 10× objective lens (Nikon, Japan). Images were captured with a camera (TV Lens C-0.6X Nikon, Japan) and acquired with Motic Images Plus 3.0 Routine Software Series.

### Cell culture and stimulation of the endocannabinoid system

SHED (1 × 10 ^5^ cells/well) were cultured in 6-well plates for 48 hours ( Table 2 ) in the presence of 5µM of anandamide (Ago; Cayman Chemical, USA), in the presence or absence of 2µM of AM251 (Ant1; Cayman Chemical) and 2µM of SR144528 (Ant2; Cayman Chemical), with or without TNF-α (10ng/mL; Sigma-Aldrich) stimulation, in supplemented DMEM/F12 at 37 ^o^ C under 5% CO _2_ . ^( 52 - 54 )^

**Table 2:** Description of the groups used for immunomodulation of stem cells from human exfoliated deciduous teeth

	TNF-α (-)	TNF-α (+)
Groups	Medium	Medium
	Anandamide (Ago)	Anandamide (Ago)
	AM251 (Ant1)	AM251 (Ant1)
	SR144528 (Ant2)	SR144528 (Ant2)
	Ant1 + Ant2 (Ant1+2)	Ant1 + Ant2 (Ant1+2)
	Ago + Ant1	Ago + Ant1
	Ago + Ant2	Ago + Ant2
	Ago + Ant1 + Ant2 (Ago+Ant1+2)	Ago + Ant1 + Ant2 (Ago+Ant1+2)

TNF-α: tumour necrosis factor alpha.

### Assessment of cell viability

Cell (500 cells/well) were plated in 96-well plates, and cultured in the presence of 5µM of anandamide, in the presence or absence of 2µM of AM251 and 2µM of SR144528, with or without TNF-α (10ng/mL) stimulation ( Table 2 ), in supplemented DMEM/F12 at 37 ^o^ C under 5% CO _2_ . After 48 hours, 10% of 0.15mg/mL resazurin sodium salt (Alamar Blue ^®^ , Thermo Fisher Scientific, USA) was added to the wells. The cells were further incubated for 4 hours and the absorbance was read at 570 and 600nm. The experiment was performed in triplicates.

### Flowcytometry analysis of cell markers

For flow cytometry analysis, cultured SHED ( Table 2 ) were washed three times with 1X PBS and enzymatically disaggregated using 0.25% trypsin (TrypLE Express, Invitrogen, USA) for five minutes at 37°C; the enzyme was inactivated by adding the culture medium. The cells were centrifuged at 300 × *g* for five minutes, counted using a hemocytometer (BD Biosciences) with blue, red, and violet laser, and labeled for the surface markers HLA-DR (V500 mouse anti-human HLA-DR; BD Bioscience), PDL-1 [BB515 mouse anti-human PDL-1, isotype: mouse (BALB/c) IgG1, clone: MIH1; BD Bioscience], and PDL-2 (APC mouse anti-human PDL-2, isotype: mouse IgG1, κ, clone: MIH18; BD Bioscience). A total of 50,000 events were acquired for each group and SHED not labeled with antibodies were used as negative control. The experiment was performed in triplicates.

### Statistical analysis

The data collected *in vitro* were analyzed with appropriate statistical tools taking into account the sample number and normal distribution, using the Shapiro-Wilk normality test. Comparisons between the means of the analyzed groups were made using the Kruskal-Wallis test, and post-hoc Dunn’s multiple comparison test. The differences were considered significant at p<0.05 for the two-tailed analysis. Statistical analyses and graphical representation of data were done using the GraphPad Prism software (version 7; GraphPad Software, Inc., USA).

## RESULTS

### Isolation, characterization, and differentiation of SHED

**Figure 1:**
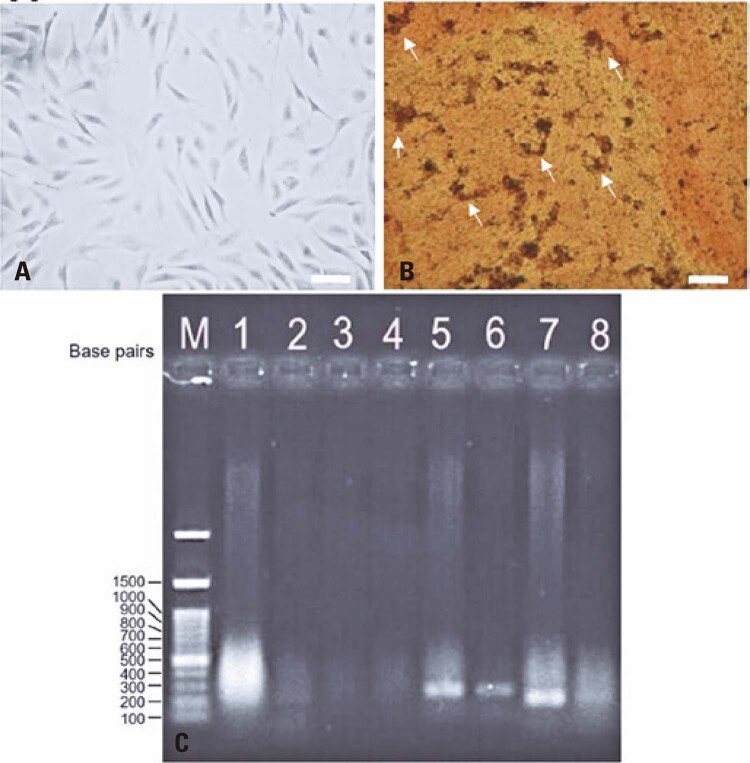
Characterization of stem cells from human exfoliated deciduous teeth (SHED). A) Fusiform morphology of SHED (×10 magnification); B) Alizarin red staining indicating osteogenic differentiation of SHED (white arrows indicate calcium deposits). The cytoplasm is stained red and extracellular calcium deposits are stained black (×4 magnification); C) Molecular characterization of mesenchymal and embryonic markers (1, *ACTB* ; 2, *APDH* ; 3, *CD34* ; 4 *CD45* ; 5, *NES* ; 6, *CD105* ; 7, *OCT4* ; 8, *NANOG* ). Scale bars: A) 200µm and B) 100µm

In basal medium, SHED showed a typical fibroblast-like appearance ( Figure 1A ). To test the osteogenic differentiation capacity, isolated cells were cultured in an osteogenic induction medium. SHED showed osteoblast-like morphology and after staining with Alizarin Red, calcium deposits were evidenced ( Figure 1B ).

The expression of typical mesenchymal and embryonic marker genes in SHED was assessed using RT-PCR. SHED expressed not only the marker genes for MSC ( *NES* and *CD105* ) but also for embryonic cells ( *NANOG* and *OCT-4* ) ( Figure 1C ). The marker genes for hematopoietic lineages ( *CD34* and *CD45* ) were not expressed, as expected. ^( 12 , 55 )^

The viability of SHED cultured in the presence or absence of agonist, antagonists, and TNF-α was not significantly different among the groups and when compared to that of cells cultured with the medium alone (data not shown).

### HLA-DR expression

**Figure 2:**
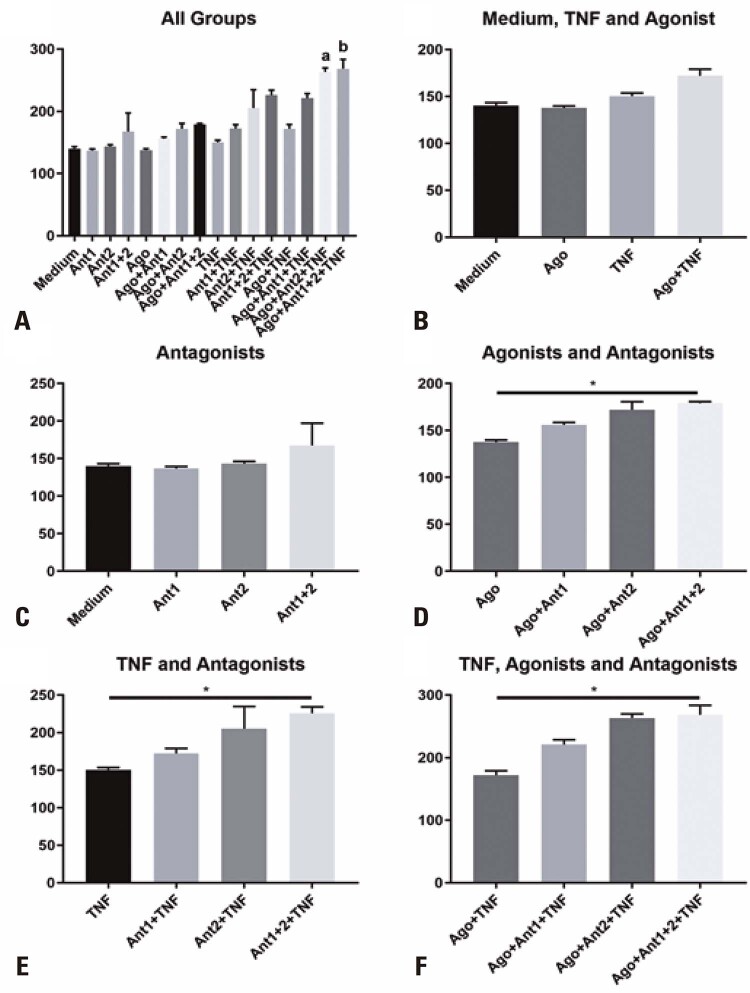
Median fluorescence intensity of human leukocyte antigen-DR isotype (HLA-DR) on the surface of stem cells from human exfoliated deciduous teeth (SHED). A) All groups compared with medium; B) SHED treated with tumor necrosis factor alpha (TNF-α), agonist, or both, or left untreated; C) SHED treated with antagonist 1, 2, or both, or left untreated; D) SHED in the presence of the agonist and treated with antagonist 1, 2, or both, or left untreated; E) SHED in the presence of TNF-α and treated with antagonist 1, 2, or both, or left untreated; F) SHED in the presence of the agonist and TNF-α and treated with antagonist 1, 2, or both, or left untreated. a - groups showing statistical difference at p<0.05 in relation to the Medium Group; b - groups showing statistical difference at p<0.01 in relation to the Medium Group * p<0.05.

Initially, all groups were compared with the group cultured only with the medium (medium; without the presence of TNF-α, agonist, and antagonists) to ascertain the situation in which SHED would have altered amounts of HLA-DR on their surfaces. Only the groups that received TNF-α, agonist, and mainly antagonist 2 (Ago+Ant2+TNF and Ago+Ant1+2+TNF) treatment showed a statistically significant difference compared with the group cultured only in the medium ( Figure 2A ). Thereafter, we analyzed whether TNF-α, agonist, or both could alter the levels of HLA-DR. Despite a slight increase in the level of this marker in SHED in the presence of TNF-α or TNF-α and agonist, there was no statistical difference between the groups ( Figure 2B ).

Subsequently, the effect of the antagonists was analyzed within the groups that received TNF-α, agonist, or their combination ( Figure 2C to F ). The presence of the antagonists alone did not lead to any change in HLA-DR levels ( Figure 2C ). However, the groups that received both the antagonists (Ant1+2) and also received the agonist ( Figure 2D ), TNF-α ( Figure 2E ) or Agonist+TNF-α ( Figure 2F ), showed a difference vis-à-vis their respective reference groups (Ago, TNF-α, and Ago+TNF-α).

### PD-L1 expression

**Figure 3:**
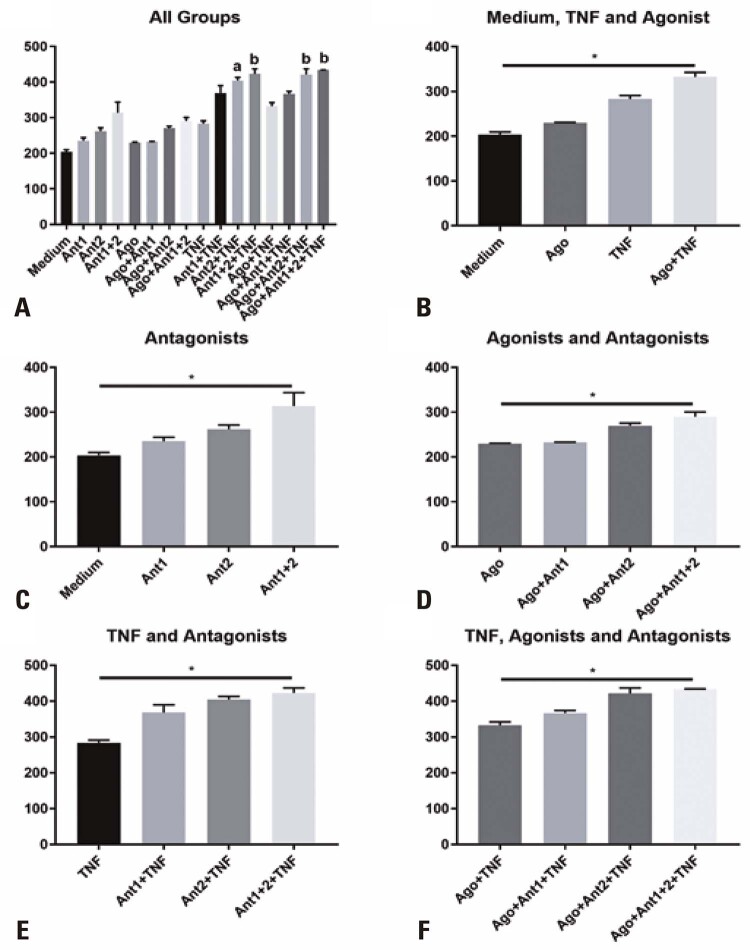
Median fluorescence intensity of programmed death ligand 1 (PD-L1) on the surface of stem cells from human exfoliated deciduous teeth (SHED). A) All groups compared with Medium; B) SHED treated with tumor necrosis factor alpha (TNF-α), agonist, or both, or left untreated; C) SHED treated with antagonist 1, 2, or both, or left untreated; D) SHED in the presence of the agonist and treated with antagonist 1, 2, or both, or left untreated; E) SHED in the presence of TNF-α and treated with antagonist 1, 2, or both, or left untreated; F) SHED in the presence of the agonist and TNF-α and treated with antagonist 1, 2, or both, or left untreated. a - groups showing statistical difference at p<0.05 in relation to the Medium Group; b - groups showing statistical difference at p<0.01 in relation to the Medium Group * p<0.05.

In the comparison of all groups with the Medium Group, the groups that received antagonist 2 or both the antagonists in the presence of TNF-α, but independent of the agonist, showed a significant difference ( Figure 3A ). When comparing the means between the Medium and Ago, TNF-α, and Ago+TNF-α Groups, the groups that received TNF-α showed an increase compared to the Medium and Ago Groups, but the differences were significant only between the Medium and Ago+TNF-α Groups ( Figure 3B ). The analysis of the role of the antagonists revealed that the groups receiving both the antagonists showed a significant difference relative to their respective reference groups-without agonist and without TNF-α ( Figure 3C ), with agonists ( Figure 3D ), with TNF-α ( Figure 3E ), or with agonists and TNF-α ( Figure 3F ).

### PD-L2 expression

**Figure 4:**
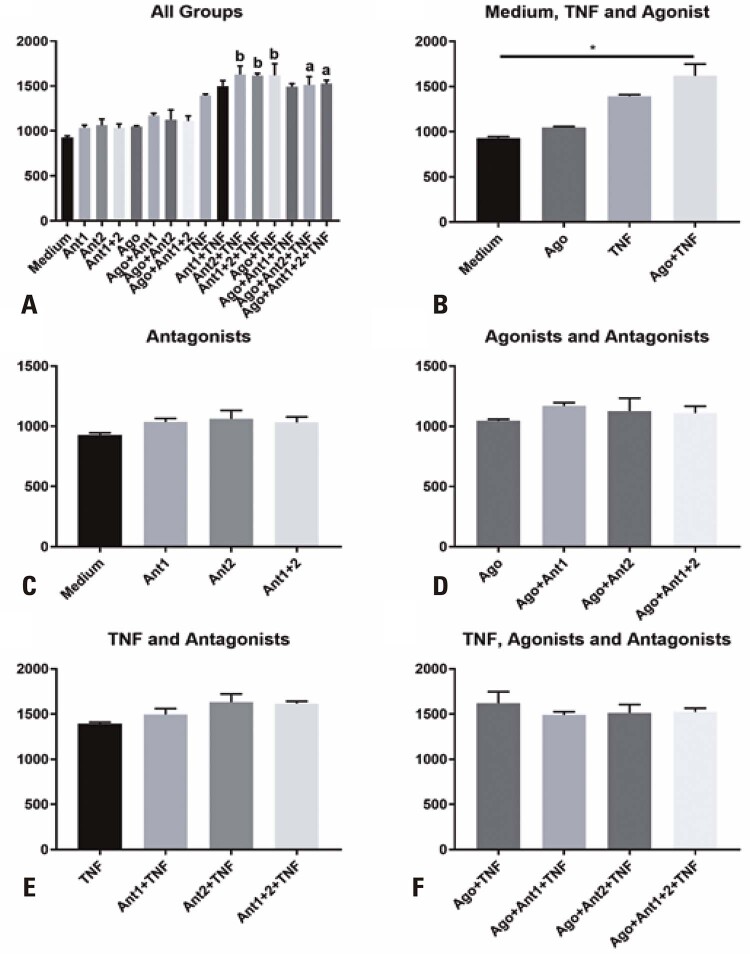
Median fluorescence intensity of programmed death ligand 2 (PD-L2) on the surface of stem cells from human exfoliated deciduous teeth (SHED). A) All groups compared with Medium; B) SHED treated with tumor necrosis factor alpha (TNF-α), agonist, or both, or left untreated; C) SHED treated with antagonist 1, 2, or both, or left untreated; D) SHED in the presence of the agonist and treated with antagonist 1, 2, or both, or left untreated; E) SHED in the presence of TNF-α and treated with antagonist 1, 2, or both, or left untreated; F) SHED in the presence of the agonist and TNF-α and treated with antagonist 1, 2, or both, or left untreated. a - groups showing statistical difference at p<0.05 in relation to the Medium Group; b - groups showing statistical difference at p<0.01 in relation to the Medium Group *p<0.05.

Similar to the levels of the other markers, PD-L2 levels were significantly increased in the groups that received antagonists 1, 2, or both, in the presence of TNF-α relative to the levels in the Medium Group; however, in the Ant1+TNF-α and Ago+Ant1+TNF-α groups, the increase in PD-L2 levels was not significant ( Figure 4A ).

SHED showed increased PD-L2 levels in the presence of TNF-α, but the increase was significant when SHED were cultured in the presence of TNF-α and the agonist ( Figure 4B ). As for the analyses for the antagonists in cells that did or did not receive agonists and TNF-α, there were no significant changes in the expression of the PD-L2 marker between the groups ( Figure 4C to F ).

## DISCUSSION

In most studies, MSCs are considered potent inhibitors of the immune system. It is believed that such property of MSCs is not fixed or constitutive, but is influenced by the inflammatory environment. The immune system should act as a mediator, regulating not only the immunomodulatory properties of MSCs but also their proliferation and differentiation, thereby, actively influencing the process of damage recovery. Some inflammatory molecules, such as TNF-α and IFN-γ, appear to be involved in this process. ^( 3 , 8 , 10 , 15 , 23 , 25 )^

Mesenchymal stromal cells apparently have an immunoregulatory role and an antigen-presenting cell (APC) role, depending on the type, intensity, and timing of the inflammatory process. At the commencement of the inflammatory process, MSCs increase their antigen-presenting potential for fighting a possible infection. However, as inflammation progresses, the environment modulates MSCs to acquire immunosuppressive potential, for restoring homeostasis of the cellular and tissue environment. ^( 5 , 9 , 10 , 56 - 62 )^

Different types of MSCs share immunomodulatory effects and have great therapeutic potential. The immunomodulatory capacity of dental pulp stem cells (DPSCs) and SHED is no exception, and these cells sometimes show superior capacity compared with traditional bone marrow MSCs (BMSCs). ^( 1 , 51 , 56 )^

With regard to the presence of the ECS outside of the CNS, CB1 has been identified in the liver, bone marrow, pancreas, lungs, vascular system, muscles, gastrointestinal tract, reproductive organs, and the immune system. ^( 45 - 47 )^ CB2 has also been detected in the immune system, mainly in B cells, natural killer cells, and monocytes, which play an active role in the modulation of cell migration and cytokine release. ^( 45 - 47 )^ Molecules involved in the ECS are produced from membrane phospholipid precursors, and exerting immunomodulatory actions, such as decreased production of proinflammatory cytokines and modulation of *T* - *helper* (Th) *1* and *Th2* (TH1/TH2) cell responsiveness. ^( 39 , 42 , 63 , 64 )^

Mesenchymal stromal cells can express all the components of the ECS. AEA, CB1, and CB2 are expressed and are involved in the physiological protection against intense inflammatory responses, with CB1 participating in the healing of inflamed tissues. Furthermore, CB1 is activated during osteogenic differentiation of BMSCs and periodontal ligament stem cells, even under an inflammatory environment. CB1 was reported to be an essential component in the survival of differentiated cells during acute stress. ^( 48 , 49 )^

The present study sought to evaluate the role of the ECS in the presence of TNF-α and to assess the possible cellular phenotypic alterations. In general, TNF-α and CB1 and CB2 receptor antagonists, especially the CB2 antagonist, were able to alter the immunological phenotype of the ECS with regard to the three markers analyzed. Because there was no significant difference in the cell viability test, any change occurring between the groups is expected to result from the alteration in the immunological phenotype of SHED.

We noted that not only the presence of TNF-α together with the antagonists (mainly of CB2) but the presence of the agonist also led to increased HLA-DR expression in SHED. In this regard, anandamide may be activating other ECS receptors, such as G protein-coupled receptor 55 (GPR55) and transient receptor potential (TRP) channels and their respective subfamilies. ^( 43 )^ These results indicate that SHED exhibit immunological response under these conditions. Palomares Cabeza et al. ^( 34 )^ showed that, under inflammatory conditions, these cells are able to interact with T lymphocytes via HLA-DR, because HLA-DR is especially responsible for initiating adaptive immunity by presenting antigens to these lymphocytes. The results of the present study are in agreement with the notion that SHED function as APCs or the with the possibility of inhibiting inflammatory processes via the presentation of specific antigens to T cells, depending on the degree of inflammation of the microenvironment. The presence of SHED in the inflammatory environment leads to decreased proliferation of Th cells and increased proliferation of Treg, ^( 9 , 56 - 58 )^ explaining the targeting/maintenance of an immunoregulatory profile.

From figures 3A and 4A , it is evident that TNF-α together with the antagonists could drive SHED toward an immunomodulatory profile both via PD-L1 and PD-L2. This result was most evident for PD-L1, in which case the blockade of CB1 and CB2 increased the levels of this marker, especially in the presence of TNF-α. Thus, the increase in PD-L1 and PD-L2 levels under these conditions corroborates with the results of studies in which the presence of SHED in an inflammatory environment could lead to immunoregulation. ^( 9 , 56 - 58 )^ Specifically, the CB1 receptor antagonist showed a slight, but nonsignificant, increase in HLA-DR and PD-L1 levels when SHED were cultured in the presence of TNF-α. The contribution of the CB2 antagonist, in the presence of TNF-α, on all three markers was evident, most notably for HLA-DR and PD-L1. However, the changes were only significant when both the antagonists were used. In this sense, the literature regarding CB1 and CB2 receptors is contradictory, with the consensus being only on the participation of the ECS in immunomodulation, but not on the role in activation and blockade of each receptor. ^( 39 , 42 , 63 , 64 )^ Galve-Roperh et al. ^( 6 )^ reported that CB2 activation is associated with chronic inflammation of the nervous system as well as with different immune disorders. Montanari et al. ^( 46 )^ described a series of novel benzofuran-based compounds with neuroprotective and immunomodulatory properties for the treatment of Alzheimer’s disease, resulting in the change of proinflammatory M1 phenotype to the neuroprotective M2 phenotype of microglia. Rossi et al. ^( 50 )^ reported that CB1 promotes inflammation while CB2 regulates its magnitude. Moreno et al. ^( 42 )^ noted that CB2 ligands are responsible for the observed immunomodulatory effects.

The ECS is present in both immune system cells and MSCs, expressing both CB1 and CB2 receptors, which is suggestive of a possible common route of immunomodulation, mainly via CB2. ^( 48 , 50 , 64 )^ In a study with endothelial progenitor cells, the release of AEA and 2-AG was verified. In the presence of TNF-α, there was an increase in 2-AG levels. Moreover, after treatment of this mature cell line with endocannabinoids, a reduction in the induction (by TNF-α) of the proinflammatory adhesion molecule CD106 (VCAM) was observed. ^( 65 )^ In addition, cannabidiol was also reported to protect oligodendrocyte progenitor cells from apoptosis induced by lipopolysaccharides (LPS)- or INF-γ-stimulated inflammation. Cannabidiol treatment reversed the induction of caspase 3, decreased the production of reactive oxygen species and endoplasmic reticulum stress-induced apoptosis, followed by decreased levels of molecular effectors, Bax and caspase 12. ^( 39 )^

The expression of CB1 receptor is increased during osteogenic differentiation of MSCs and it is essential for the survival of these differentiated cells. ^( 48 )^ Tetrahydrocannabinol (THC) was able to activate the CB2 receptor, increasing BMSC immunoregulation of the release of inflammation-associated cytokines (TNF-α, interleukin [IL]-1β, IL-6, and IL-8). Furthermore, hyperalgesia and allodynia were significantly reduced when MSCs pretreated with THC were administered; consequently, the levels of proinflammatory cytokines were also significantly reduced, whereas IL-10 levels were increased. ^( 66 )^ It was shown that CB2 activation in MSCs led to increased IL-10 release and reduced levels of LPS-induced proinflammatory cytokines, IL-1β, IL-8, and IL-17. ^( 50 )^

In a study analyzing the molecular phenotype of gingival mesenchymal stromal cells (GMSCs), treatment with cannabidiol was verified to prevent the expression of genes of the NALP3-inflammasome pathway, suppressing the levels of *NALP3* , *CASP1* , and *IL-18* , demonstrating the inhibition of apoptosis, accompanied by the suppression of Bax. Cannabidiol treatment could also reduce the expression of genes involved in the activation of the immune system ( *CD109* , *CD151* , *CD40* , *CD46* , *CD59* , *CD68* , *CD81* , *CD82* , *CD99* ), while stimulating the expression of genes involved in the inhibition of immune responses ( *CD47* , *CD55* , *CD276* ) Libro et al. ^( 67 )^ In another study, pretreatment of GMSCs with cannabidiol led to the attenuation of the expression of genes implicated in the etiopathogenesis of Alzheimer’s disease, showing that preconditioned GMSCs have potential for the treatment of early-stage Alzheimer’s disease. ^( 67 )^

The findings of the present work lend support to the hypothesis that SHED have an immunomodulatory role in an environment with the presence of inflammatory molecules, and may act directly together with cells of the immune system in coordinating the inflammatory process by inhibiting the adaptive response via PD-L1 and PD-L2, possibly occurring in conjunction with the HLA-DR and TCR interaction, leading to lymphocyte tolerance. ^( 27 - 29 , 34 , 68 )^

## CONCLUSION

The inhibition of the endocannabinoid receptors together with the proinflammatory effect of TNF-α led to an increased expression of HLA-DR on the surface of stem cells from human exfoliated deciduous teeth, and also conferred an anti-inflammatory profile on these cells by increasing the PD-L1 and PD-L2 levels. The agonist treatment also influenced the alteration of these surface proteins; however, we could not show the exact modulation of the immune profile of stem cells from human exfoliated deciduous teeth because the endocannabinoid system is a complex system and involves other receptors that were not analyzed in this study. Nonetheless, this study contributes to broadening the investigation of the interaction of stem cells from human exfoliated deciduous teeth and the endocannabinoid system in relation to immunomodulation. The interactions among the mesenchymal stromal cells, immune system, and the endocannabinoid system revealed in this study highlight the importance of investigating the cellular and physiological effects of active principles that act on the endocannabinoid system for the elucidation and demystification of their actions and consequences in the treatment of diseases.
